# Supplementary material for: From sequence to enzyme mechanism using multi-label machine learning
Source: BMC Bioinformatics. 2014 May 19;15:150. doi: 10.1186/1471-2105-15-150 (PMC4229970; doi:10.1186/1471-2105-15-150)
Supplement: Additional file 2 — Java code of ml2db. Additional file ml2db_code.tar.gz contains the Java source code to run the multi-label machine learning experiments and save the results to database. The code’s Javadoc is included. [file 1471-2105-15-150-S2.zip › additional file 2/ml2db/utils/doc/uk/ac/standrews/utils/main/maputils/MapUtils.html]

MapUtils


JavaScript is disabled on your browser.


- Overview
- Package
- Class
- Use
- Tree
- Deprecated
- Index
- Help

- Prev Class
- Next Class

- Frames
- No Frames

- All Classes

- Summary:
- Nested |
- Field |
- Constr |
- Method

- Detail:
- Field |
- Constr |
- Method


uk.ac.standrews.utils.main.maputils

## Class MapUtils

- java.lang.Object
- - uk.ac.standrews.utils.main.maputils.MapUtils

- ---

    

  ```
  public class MapUtils
  extends java.lang.Object
  ```

  Utilities to manipulate maps and hashmaps

  Version:
  :   4 Jan 2008

  Author:
  :   Luna De Ferrari luna.deferrari-at-ed.ac.uk

- - ### Field Summary

    Fields

    | Modifier and Type | Field and Description |
    | --- | --- |
    | `static boolean` | `M_DEBUG` |
  - ### Constructor Summary

    Constructors

    Constructor and Description

    `MapUtils()`
  - ### Method Summary

    Methods

    | Modifier and Type | Method and Description |
    | --- | --- |
    | `static java.util.TreeMap<java.lang.Integer,java.lang.String>` | `addAll(java.util.TreeMap<java.lang.Integer,java.lang.String> map1, java.util.TreeMap<java.lang.Integer,java.lang.String> map2)` Add all key-value entries of map2 into map1 and return map1 |
    | `static java.util.Map.Entry` | `extractMapEntry(java.util.Map map, int i)` Extracts a random entry (key-value association) from a map |
    | `static java.util.TreeMap` | `flipMap(java.util.TreeMap map)` Substitutes keys and values in a map. |
    | `static <T> OneToManyMap<T,T>` | `fromArrayOfArrayToOneToManyMap(T[][] array)` Array format must be [[key1, value1][key1, value2],[key2, value3],[...]]. |
    | `static java.util.TreeMap<java.lang.String,java.lang.String>` | `fromTwoVectorsToMap(java.util.Vector<java.lang.String> keys, java.util.Vector<java.lang.String> values)` Creates an hashmap from 2 vectors. |
    | `static double` | `getDoubleFromHashmap(java.util.HashMap hashMap, java.lang.String hashMapKey)` Gets a double from a hashMap |
    | `static java.util.ArrayList<java.lang.String>` | `getFirstValueOccurrence(java.util.Map<java.lang.String,java.lang.String> map, java.lang.String value)` Get all the keys that have the given value in a Map\ |
    | `static java.lang.String` | `getHighestValuedKey(java.util.TreeMap<java.lang.String,java.lang.Double> map)` Return the key with the highest value (or the first key with the highest value, if many have the same value). |
    | `static java.lang.String` | `getHighestValuedKeyRandomly(java.util.TreeMap<java.lang.String,java.lang.Double> map)` Return the key with the highest value (or one key \*at random\* among the ones with the highest value). |
    | `static java.util.Map<java.lang.Integer,java.util.Map.Entry>` | `getIndexedMap(java.lang.Object[] mapEntries)` Get a map with entries composed of: an integer id as key and an entry as value |
    | `static boolean` | `mapIsNullOrEmpty(java.util.Map map)` |
    | `static java.lang.String` | `mapToString(java.util.Map map)` Creates a string representation of a map, as index,tab,key,tab,value |
    | `static void` | `printMap(java.util.Map map)` Prints an HashMap as a list of index + key + value |
    | `static java.util.TreeMap<java.lang.String,java.lang.String>` | `removeEmptyKeys(java.util.TreeMap<java.lang.String,java.lang.String> map)` |
    | `static boolean` | `treeMapsHaveEqualValues(java.util.TreeMap<java.lang.Integer,java.lang.Character> map1, java.util.TreeMap<java.lang.Integer,java.lang.Character> map2)` Check if two treeMaps have the same collection of values |

    - ### Methods inherited from class java.lang.Object

      `equals, getClass, hashCode, notify, notifyAll, toString, wait, wait, wait`

- - ### Field Detail


    - #### M\_DEBUG

      ```
      public static boolean M_DEBUG
      ```
  - ### Constructor Detail


    - #### MapUtils

      ```
      public MapUtils()
      ```
  - ### Method Detail


    - #### flipMap

      ```
      public static java.util.TreeMap flipMap(java.util.TreeMap map)
      ```

      Substitutes keys and values in a map. use at your own risk, the values \*must\* be unique or data can be lost...


    - #### extractMapEntry

      ```
      public static java.util.Map.Entry extractMapEntry(java.util.Map map,
                                        int i)
      ```

      Extracts a random entry (key-value association) from a map

      Parameters:
      :   `map` - a map

      Returns:
      :   a random (truncated Pareto distributed) entry from the map


    - #### fromArrayOfArrayToOneToManyMap

      ```
      public static <T> OneToManyMap<T,T> fromArrayOfArrayToOneToManyMap(T[][] array)
      ```

      Array format must be [[key1, value1][key1, value2],[key2, value3],[...]].
      Output map format: key1 {value1, value2}, key2 {value}, ...

      Parameters:
      :   `array` -

      Returns:
      :   a one to many map


    - #### fromTwoVectorsToMap

      ```
      public static java.util.TreeMap<java.lang.String,java.lang.String> fromTwoVectorsToMap(java.util.Vector<java.lang.String> keys,
                                                                             java.util.Vector<java.lang.String> values)
      ```

      Creates an hashmap from 2 vectors. The first vector must contain a the
      keys, the second vector the values. The keys and values vectors must have
      the same size.

      Parameters:
      :   `keys` -
      :   `values` -

      Returns:
      :   an `HashMap`


    - #### getDoubleFromHashmap

      ```
      public static double getDoubleFromHashmap(java.util.HashMap hashMap,
                                java.lang.String hashMapKey)
      ```

      Gets a double from a hashMap


    - #### getFirstValueOccurrence

      ```
      public static java.util.ArrayList<java.lang.String> getFirstValueOccurrence(java.util.Map<java.lang.String,java.lang.String> map,
                                                                  java.lang.String value)
      ```

      Get all the keys that have the given value in a Map\

      Parameters:
      :   `map` - a map
      :   `value` - a hash map value

      Returns:
      :   the keys


    - #### getHighestValuedKey

      ```
      public static java.lang.String getHighestValuedKey(java.util.TreeMap<java.lang.String,java.lang.Double> map)
      ```

      Return the key with the highest value (or the first key with the highest
      value, if many have the same value).

      Parameters:
      :   `map` - a map with a string key and a double value

      Returns:
      :   the key with the highest value (or the first key with the highest
          value, if many have the same value).


    - #### getHighestValuedKeyRandomly

      ```
      public static java.lang.String getHighestValuedKeyRandomly(java.util.TreeMap<java.lang.String,java.lang.Double> map)
      ```

      Return the key with the highest value (or one key \*at random\* among the
      ones with the highest value).

      Parameters:
      :   `map` - a map with a string key and a double value

      Returns:
      :   the key with the highest value (or one key \*at random\* among the
          ones with the highest value).


    - #### getIndexedMap

      ```
      public static java.util.Map<java.lang.Integer,java.util.Map.Entry> getIndexedMap(java.lang.Object[] mapEntries)
      ```

      Get a map with entries composed of: an integer id as key and an entry as
      value

      Parameters:
      :   `mapEntries` - an array of Entry objects

      Returns:
      :   a map with entries composed of: an integer id as key and an entry
          as value


    - #### mapIsNullOrEmpty

      ```
      public static boolean mapIsNullOrEmpty(java.util.Map map)
      ```


    - #### mapToString

      ```
      public static java.lang.String mapToString(java.util.Map map)
      ```

      Creates a string representation of a map, as index,tab,key,tab,value

      Returns:
      :   a string representation of the map


    - #### printMap

      ```
      public static void printMap(java.util.Map map)
      ```

      Prints an HashMap as a list of index + key + value

      Parameters:
      :   `map` -


    - #### removeEmptyKeys

      ```
      public static java.util.TreeMap<java.lang.String,java.lang.String> removeEmptyKeys(java.util.TreeMap<java.lang.String,java.lang.String> map)
      ```


    - #### treeMapsHaveEqualValues

      ```
      public static boolean treeMapsHaveEqualValues(java.util.TreeMap<java.lang.Integer,java.lang.Character> map1,
                                    java.util.TreeMap<java.lang.Integer,java.lang.Character> map2)
      ```

      Check if two treeMaps have the same collection of values

      Parameters:
      :   `map1` -
      :   `map2` -

      Returns:
      :   true, if the two treeMaps have the same collection of values


    - #### addAll

      ```
      public static java.util.TreeMap<java.lang.Integer,java.lang.String> addAll(java.util.TreeMap<java.lang.Integer,java.lang.String> map1,
                                                                 java.util.TreeMap<java.lang.Integer,java.lang.String> map2)
      ```

      Add all key-value entries of map2 into map1 and return map1

      Parameters:
      :   `map1` -
      :   `map2` -


- Overview
- Package
- Class
- Use
- Tree
- Deprecated
- Index
- Help

- Prev Class
- Next Class

- Frames
- No Frames

- All Classes

- Summary:
- Nested |
- Field |
- Constr |
- Method

- Detail:
- Field |
- Constr |
- Method
